# The influence of social preferences and reputational concerns on intergroup prosocial behaviour in gains and losses contexts

**DOI:** 10.1098/rsos.150546

**Published:** 2015-12-23

**Authors:** Jim A. C. Everett, Nadira S. Faber, Molly J. Crockett

**Affiliations:** Department of Experimental Psychology, University of Oxford, Oxford, UK

**Keywords:** prosocial behaviour, ingroup favouritism, loss aversion, reputation, preferences

## Abstract

To what extent do people help ingroup members based on a social preference to improve ingroup members’ outcomes, versus strategic concerns about preserving their reputation within their group? And do these motives manifest differently when a prosocial behaviour occurs in the context of helping another gain a positive outcome (study 1), versus helping another to avoid losing a positive outcome (study 2)? In both contexts, we find that participants are more prosocial towards ingroup (versus outgroup members) and more prosocial when decisions are public (versus private) but find no interaction between group membership and either anonymity of the decision or expected economic value of helping. Therefore, consistent with a preference-based account of ingroup favouritism, people appear to prefer to help ingroup members more than outgroup members, regardless of whether helping can improve their reputation within their group. Moreover, this preference to help ingroup members appears to take the form of an intuitive social heuristic to help ingroup members, regardless of the economic incentives or possibility of reputation management. Theoretical and practical implications for the study of intergroup prosocial behaviour are discussed.

## 1. Introduction

Prosocial behaviour—the performance of acts beneficial to other people—is a central feature of human social life. In particular, people tend to act more prosocially towards ingroup members than outgroups: a phenomenon known as ingroup favouritism or ingroup-favouring prosocial behaviour. Ingroup favouritism has been consistently demonstrated in a number of experimental paradigms [1–11], even in non-interdependent contexts where prosocial behaviour cannot be directly reciprocated. But what psychological mechanisms drive ingroup-favouring prosocial behaviour? Do people help ingroup members based on a social preference to enhance ingroup members’ outcomes, or because of strategic concerns about preserving their reputation within their group? And do these motives manifest differently when a prosocial action occurs in the context of helping another gain a positive outcome, versus helping another to avoid losing a positive outcome? In two experiments, we studied these questions, investigating the extent to which ingroup favouritism is driven by social preferences versus reputational concerns, in both gains and losses contexts.

## 2. Social preferences and reputational concerns

Two accounts dominate psychological explanations of ingroup favouritism [12,13]. On the one hand is the largely preference-based social identity approach generally favoured in traditional social psychology [14,15], while on the other is the largely belief-based theory of *bounded generalized reciprocity* (BGR) focusing on reciprocity and reputational concerns generally favoured in evolutionary psychology [16,17].

The social identity approach suggests that ingroup favouritism is a kind of *preference*—i.e. people place a higher positive value on the outcomes of ingroup members, relative to outgroup members [13,14]. Preferences refer to a person’s dispositions towards certain behaviours and the outcomes based on the utility expected to be derived from them, and *social preferences* in particular refer to those other-regarding preferences concerning the well-being of others, fairness and reciprocity [18–21]. Put simply, preference-based accounts of ingroup favouritism hold that, for a variety of reasons, people like to help ingroup members more than outgroup members. According to a social identity preference-based account, identifying with a group leads to ingroup favouritism because the individual’s own interests become more aligned with the interests of the group collective, thus enhancing the desire to help ingroup members—just as one would want to help oneself [14,15]. While a strong preference-based approach privileges an analysis of expected utility to be obtained from helping, it is important to note that a weaker form of social preferences might more closely approximate *social heuristics*, whereby behaviours that have promoted utility in the past become internalized and automatic [13,22,23].

In contrast, a reputation-based account—such as the BGR—posits that ingroup favouritism is primarily driven not by greater preferences for helping ingroup members, but rather by selective strategic concerns about the preservation of a positive reputation with ingroup members. Evolutionary accounts positing a reputation-based theory of cooperation in groups (*indirect reciprocity*) hold that through being helpful in situations where others know that the helper will not benefit directly, the person builds a reputation of trustworthiness, thus enhancing their evolutionary fitness [24–26]. Indeed, reputation building is an important factor in explaining general prosocial behaviour in economic games [27,28]. With regards to intergroup prosocial behaviour specifically, it has been argued that reputational concerns are of primary importance in causing ingroup favouritism because group members are strategically and selectively concerned with signalling a positive reputation towards other ingroup members [17,29–31]. According to the BGR approach of Yamagishi and colleagues, group membership alone is insufficient to engender ingroup favouritism, and instead such behaviour occurs only when group members believe that other ingroup members can, or will, reciprocate in turn [29]. A related perspective suggests that people act more prosocially towards ingroup members because they perceive such ingroup-favouring behaviour to be the socially approved form of action and are aware of the reputational costs of violating their ingroup’s norms [32,33].

To what extent do these distinct motives—social preferences and reputational concerns—explain ingroup favouritism? Unfortunately, existing evidence that directly compares these motives—using, for example, *dictator games* (DGs) [34]—is inconclusive. Some studies show that ingroup favouritism is observed in DGs only when dictators can be identified as ingroup members by the recipient, and not when decisions are private, suggesting that reputational concerns are necessary for the behavioural expression of favouritism [30,31]. However, other studies show that ingroup favouritism is observed even in anonymous DGs where reputational concerns are eliminated, suggesting ingroup-favouring behaviour can be driven by social preferences alone [35–37]. Finally, some studies show that ingroup favouritism is observed under anonymity, but is considerably stronger when participants can be identified, suggesting that both social preferences and reputational concerns play an important role [38].

Despite its clear strengths (e.g. careful manipulation of whether decisions are seen by other participants or not), prior work on this topic suffers from a few potential limitations. For example, previous studies have used one-shot between-subject DGs, where participants make a single decision about whether to transfer money to either an ingroup or outgroup member, under conditions of either anonymity or not [31,38]. Such designs preclude the possibility of looking at the relationship between preference-based and reputation-based ingroup favouritism within the same participants. Furthermore, most past research has used one-trial DGs where helping has a fixed cost, and while some evidence suggests that ingroup favouritism is sensitive to costs [39], it remains unknown whether the psychological mechanisms driving ingroup favouritism are themselves sensitive to the cost of helping. We sought to address these issues in our studies.

We followed the basic design of previous work by having individuals make either public or private prosocial decisions towards ingroup or outgroup members. At the same time, however, we made a number of methodological improvements. First, we complemented previous work using a between-subjects design by using a within-subjects approach. Beyond the general benefits of within-subjects designs, particularly in economic games (e.g. reduced variance; control for individual differences across populations) [40], our within-subjects task allowed us to test the extent to which preference-based and reputation-based favouritism represent complementary, or competing, processes within participants. Second, varying the cost of helping and the benefit to the recipients across multiple trials allowed us to investigate whether concerns for economic efficiency moderate the manifestation and mechanisms of ingroup favouritism.

## 3. Gains and losses contexts in prosocial behaviour

How might intergroup prosocial behaviour manifest differently in a *gains context*—helping another gain a good outcome—versus a *losses context*—helping another avoid a bad outcome? Work on loss aversion suggests that people are more sensitive to losses than gains for the self [41] and that because inflicting a loss is seen as more harmful and fairness-violating than withholding a gain, individuals are more likely to help another avoid experiencing a harmful outcome than they are to help provide a positive outcome [42–46]. Yet no work has directly compared the effects of gains and losses on intergroup prosocial behaviour specifically. Will ingroup favouritism be observed in both gains and losses contexts? One may reason that because people are more motivated by fairness in losses contexts, they should show no preference for ingroup or outgroup members. However, we consider this hypothesis to be implausible because it assumes that greater fairness concerns in losses contexts are equal with regards to ingroup and outgroup members. In fact, evidence suggests that fairness concerns are actually more salient in interactions with ingroup members [47,48], implying that ingroup favouritism would occur in both losses and gains contexts.

Assuming that ingroup favouritism is likely to manifest in both contexts, a second question arises: to what extent is ingroup favouritism in losses contexts driven by the same psychological mechanisms as in gains contexts? Because losses are perceived as more harmful than gains, people’s social preferences to enhance the welfare of ingroup members could be more potent in driving ingroup favouritism in losses contexts. Alternatively, reputational concerns could increase ingroup favouritism in losses contexts because fairness principles are seen as more important in losses contexts and in interactions with ingroup members, and so violating such fairness principles in public towards ingroup members is likely to be more damaging to one’s reputation.

## 4. Present research

In the present research, we aimed to address two questions crucial to understanding intergroup prosocial behaviour: to what extent is ingroup favouritism driven by social preferences versus reputational concerns, in both gains (study 1) and losses contexts (study 2)?

To investigate the extent to which both social preferences and reputational concerns drive ingroup favouritism, we measured the degree to which subjects were willing to spend money to help ingroup and outgroup members, manipulating the decision setting such that these decisions were public (thus activating reputational concerns) or private (thus eliminating the potential for reputation building so that behaviour can be attributed primarily to social preferences). To the extent that social preferences drive ingroup favouritism, such ingroup-favouring prosocial behaviour should be evident regardless of decision setting—that is, whether or not decisions are public (cf. [14,35]). Considering the extent that reputational concerns drive ingroup favouritism, either a ‘strong’ or ‘weak’ prediction based on previous work can be discerned. On the strong version derived from the work of Yamagishi and Mifune, reputational concerns dominate and so ingroup favouritism should be observed ‘only when the [actor’s] behaviour is known to the in-group recipient who identifies the dictator as a member of her own group’ [31], p. 16. In other words, ingroup favouritism should *not* be observed when decisions are private and so the recipient does not know the membership or decision of the actor (cf. [31]). Alternatively, on a weaker version, reputational concerns will drive ingroup favouritism alongside social preferences, such that ingroup favouritism is observed in private conditions, but is stronger in public conditions (cf. [38]).

## 5. Method

### 5.1 Participants

Eighty participants studying at a British University were recruited for the two studies. Twenty-one participants were excluded from data analysis if they guessed hypotheses and/or the occurrence of deception in the study, or if technical problems led to unusable data. Therefore, the final sample used for analyses consisted of 29 participants in study 1 and 30 participants in study 2 (study 1, *N*=29, 15 females, *M*_age_=23, s.d.=4.45; study 2, *N*=29, 17 females, *M*_age_=21, s.d.=1.89). A power analysis revealed that for each study, 24 participants were required to detect a moderate effect size ($\eta_{p}^{2}=0.10$) at the 5% level with 80% chance, and so both of our studies were sufficiently powered.

### 5.2 Design

We report two parallel studies in this paper where we manipulated whether the recipient of the prosocial action was an ingroup or outgroup member (‘Group Membership’) and whether the prosocial decisions were anonymous or not (‘Decision Setting’). Therefore, both studies had a 2 (Group: Ingroup versus Outgroup)×2 (Decision Setting: Public versus Private) within-subjects design where participants decided whether they would pay some of their own money in the task to give a second player a better chance of earning more money. As described in detail below, the prosocial action took place either in a gains context (study 1) or a losses context (study 2). Studies 1 and 2 were distinct studies and run at separate times, but we report them in parallel in the interests of conciseness because the design and analyses for both studies were identical.

### 5.3 Procedure

Participants were recruited in groups of four. They were shown to the testing room, given an information sheet about the study and gave written consent to participate. Immediately thereafter, participants were asked to draw a small slip of paper from a hat but to refrain from looking at it until they were in their own private cubicle. The study consisted of three stages: a minimal group induction, a lottery task and a debriefing questionnaire.

#### 5.3.1 Minimal group induction

As in the work of Yamagishi & Mifune [31], we assigned participants to groups using a classic minimal group induction procedure based on preferences for the artists Klee or Kandinsky [5]. Participants were told that they would be taking part in a study on art and social preferences, and that their first task would be to judge some artworks. In individual cubicles, participants were presented with six pairs of images and were required to indicate which of each pair they preferred. Participants were told that they would be categorized into one of two groups based on their art preferences. In actuality, all participants were categorized into ‘Group A’. When participants received their group membership information, the first picture that they chose was displayed on the screen to increase the believability of the group categorizations. Participants were then asked to read a description of their group and indicate two examples of how that description was relevant to them in their everyday lives.

#### 5.3.2 Intergroup lottery task

After group categorization and still in their individual cubicles, participants were asked to read the slip of paper they had drawn from the hat at the beginning of the study. Participants were told that these slips of paper assigned them to different roles in the upcoming task. In actuality, the slips of paper were identical and all participants were told that they had been assigned to play Task 1 in the role of ‘Decider’ and would interact with two of the other participants in that testing session, who had been assigned the role of ‘Receiver’. Participants were told that they had a starting payment of £18, while the receivers would have a lower starting payment (£5 in study 1; £15 in study 2), and that their own starting payment was higher due to them making the decisions in the task. Participants were told that all three other participants were taking part in an unrelated study, but only two of the participants had the potential to receive money in the task.

The basic design of the task was as follows. Participants completed 40 trials where they made a choice between two lotteries that could win (study 1) or lose (study 2) money for a receiver. Both lotteries were always displayed on the same screen and differed in the probability of the receiver winning money, with 10 trials for each condition in a random order for each participant. The amount of money at stake was either £10 or £5, evenly split across trials. One of the lotteries was always better, in terms of having a higher expected value (EV—calculated by multiplying the size of the stake with the probability of the outcome), which meant the receiver would have a greater chance of ending up with more money. For some trials, it was free to choose the better lottery. For other trials, participants had to pay either £1 or £3 from their own starting payment of £18 in order to choose the better lottery. Participants were informed that one of the 40 trials would be randomly chosen at the end of the experiment and the decisions they chose for that round would be played and money distributed accordingly. Participants were instructed to therefore play each individual round as if it were the chosen lottery. To ensure that participants fully understood the lottery task, instructions were first presented in writing, then again orally by the experimenter, who ensured that all instructions and the task were understood and answered the participants’ questions.

**Figure 1.**
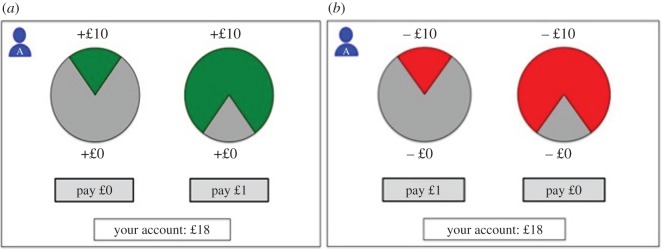
(*a*) Example decision screens for the gains-context lottery task (study 1). The figure shows that on a given trial, participants might have a choice between a left-hand lottery where they pay £0 from their own money to give another player a 20% chance of winning £10, or the right-hand lottery where they pay £1 and the other player has an 80% chance of winning £10. (*b*) Example decision screens for the losses-context lottery task (study 2). On a given trial, participants might have a choice between a left-hand lottery where they pay £1 from their own money to give another player only a 20% chance of losing £10, or the right-hand lottery where they pay nothing and the other player has an 80% chance of losing £10.

In study 1—the gains context—participants paid money in order for the other player to have a higher chance to gain more money (figure 1*a*). In study 2—the losses context—participants paid money in order for the other player to have a reduced chance of losing money (figure 1*b*). Note that the probabilities, cost for the participants and money for the other player were consistent across studies 1 and 2, with the only difference being whether this choice was in the context of helping the other player gain money, or avoid losing money.

Participants made decisions in four experimental conditions derived from a 2 (Group: Ingroup versus Outgroup)×2 (Decision Setting: Public versus Private) within-subjects design. To test whether the incidence of prosocial behaviour would be greater for ingroup or outgroup members, participants were told that they would play with two receivers: one from their own group (Group A) and one from the other group (Group B). In each trial, an icon in the top left-hand corner of the screen indicated whether the receiver for that round was an ingroup or an outgroup member (figure 1*a*,*b*). In addition, to test the roles of preferences versus reputational concerns, for half the trials participants were told that decisions were public (meaning that if that trial were selected at the end, the receiver would learn the decision and group membership of the participant), and half the trials were private (meaning that the receiver would not find out about the outcome of the lottery—or even that the deciders had been playing a game that would determine their payment for the study). Public trials were intended to raise reputational concerns, whereby participants could be motivated by the desire to preserve a positive reputation, while conversely, private trials were included to eliminate such reputational concerns because decisions were anonymous and reputation management impossible. For private trials, the icon in the left-hand corner had a blindfold, while for public trials there was no blindfold. To ensure anonymity for the private trials, participants were made aware that during the task, the receivers would not know what the deciders were doing and were completing a different task in their own cubicles. Participants were told that receivers would only learn about the lottery task if a public trial was selected at the end of the task. Participants were given a short series of questions to ensure they understood the task and the different trials, and a legend of these symbols was printed and placed next to the computer.

Our dependent measure was a binary variable indicating whether or not participants chose the better lottery on each trial. We independently varied the probabilities of the lottery to win or lose money (e.g. 25%), the sum to be earned or lost by the receiver (e.g. £10), and the cost to the participant for helping (e.g. £3), which resulted in variation, across trials, of the net expected value (net EV) associated with choosing the helpful option. Net EV is calculated by dividing the cost of choosing the better lottery from the gain in EV resulting from choosing the better lottery. For example, on trials with a low net EV, participants would have to pay a high cost (e.g. £3) to achieve a relatively small gain in EV (e.g. switching from a 10% chance of winning £5 to a 20% chance of winning £5). On trials with a high net EV, participants would have to pay a small cost (e.g. £1) to achieve a relatively large gain in EV (e.g. switching from a 10% chance of winning £10 to a 90% chance of winning £10). Excluding trials where the cost of choosing the better lottery was free, net EV took the form of three values: 1.00, 1.33 and 3.00. We then recoded these three values of net EV as three non-numeric predictors indicating low, medium and high EV. Coding this variable categorically, as opposed to continuously, allowed us to test for nonlinear as well as linear effects of net EV.

Prosocial behaviour, or helping, was defined as participants paying some of their own money to give the receiver a better chance of winning (study 1) or a lower chance of losing (study 2), and rates of helping were averaged across trials for each of the four conditions.

#### 5.3.3 Post-task questionnaire

After completing the lottery task, participants completed a final debriefing questionnaire that included demographic information and questions assessing the extent to which they had understood the task and believed the deception. Participants were asked: ‘What do you think was the purpose of this study?’; ‘During this experiment, do you think we deceived you about anything?’; and ‘Have you heard of the Tajfel (Klee Kandinsky) minimal group studies before?’. These questions were presented in the order listed, and on different pages so that the specific question on the Tajfel minimal group studies could not bias earlier responses on the first two questions. Participants who reported disbelieving the experimental instructions and paradigm were subsequently excluded, with the most common—and important—reasons for exclusions being guessing that all participants were deciders, or that the groups were not real. After the experiment was finished, it was made clear to participants that their decisions did not influence the pay-offs of other participants and that other participants would not know their decisions.

### 5.4 Analysis

In both studies, we analysed the data using repeated-measures ANOVAs and the generalized estimating equation (GEE) technique [49,50]. The GEE procedure extends a generalized linear model to allow for analysis of repeated measurements. This technique allowed us to estimate a binary dependent variable (helping) in a fully within-subject factorial design. In this model, we specified two categorical predictors (group condition; decision setting), and the categorical predictor of low, medium and high net EV (the extent to which choosing the better lottery increased the expected value of the receiver’s outcome, minus the cost to the participant; categorized as low, medium and high). The dependent measure of helping was a binary response, indicating whether participants chose the better lottery for the receiver or not.

## 6. Results

**Figure 2.**
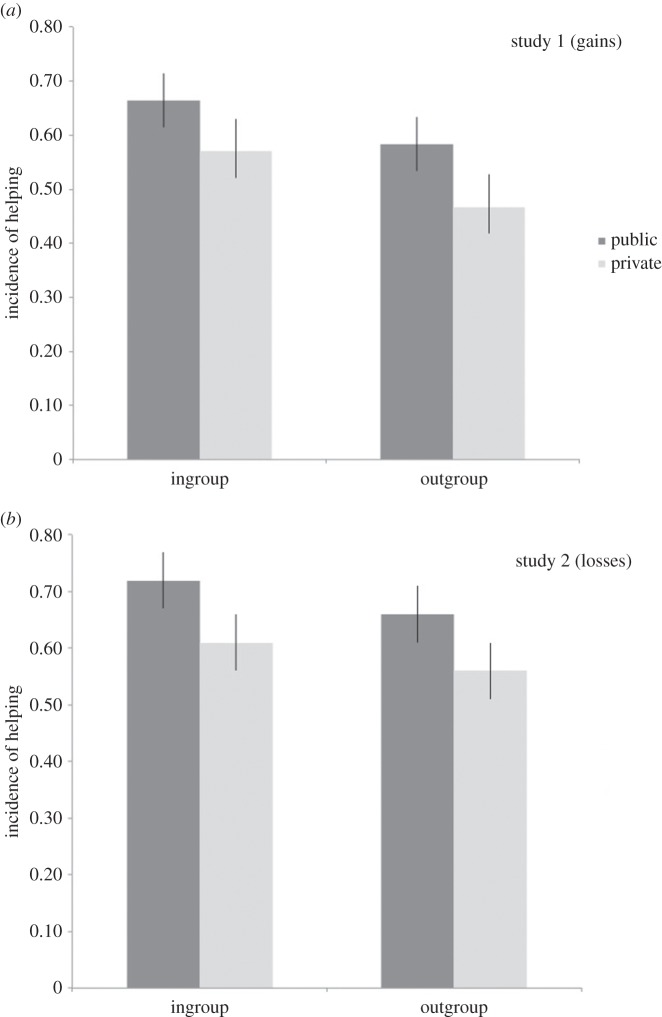
(*a*) Incidence of prosocial behaviour as a function of conditions in study 1. Results show that participants paid money to help other players gain money when the other player was an ingroup member, and when the decision was public. (*b*) Incidence of prosocial behaviour as a function of conditions in study 2. Results show that participants paid money to help other players avoid losing money when the other player was an ingroup member, and when the decision was public.

**Figure 3.**
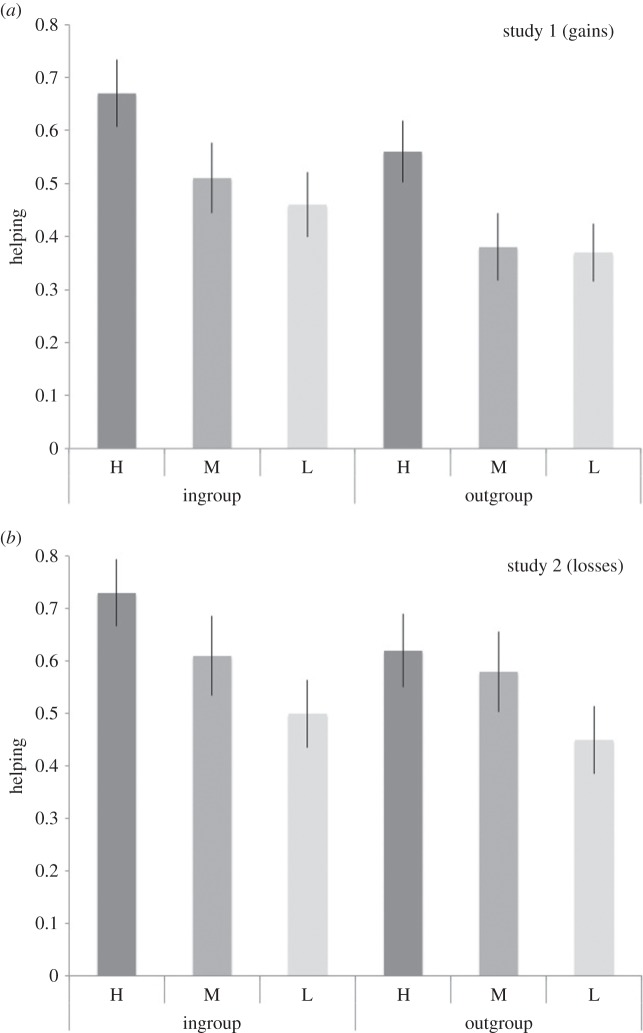
(*a*) Participants were more prosocial to ingroup than outgroup members, but there were also lower rates of helping as expected economic value decreased. (*b*) Participants were more prosocial to ingroup than outgroup members, but there were also lower rates of helping as expected economic value decreased.

We first looked at rates of helping—the extent to which participants paid some of their own money to benefit the receiver—in a repeated-measures ANOVA (Group: Ingroup versus Outgroup; Decision Setting: Public versus Private; see table 1 for all means and standard deviations). Overall, results revealed helping in about 60% of trials, with slightly higher overall rates of helping in the losses context (study 2, *M*=0.64, s.d.=0.28) than in the gains context (study 1, *M*=0.57, s.d.=0.23). In study 1, there was significant main effect of group membership such that participants were more prosocial towards ingroup members than outgroup members (*F*_1,28_=6.36, *p*=0.02, $\eta_{p}^{2}=0.19$). In study 2, the same pattern was observed, where this was marginally significant (*F*_1,29_=3.99, *p*=0.055, $\eta_{p}^{2}=0.12$). There was a main effect of decision setting in both studies (study 1, *F*_1,28_=7.74, *p*=0.01, $\eta_{p}^{2}=0.22$; study 2, *F*_1,29_=16.89, *p*<0.001, $\eta_{p}^{2}=0.37$), such that participants were more prosocial in public decisions than private decisions (figures 2 and 3). Considering the relative effect sizes, it seemed that decision setting had a stronger effect on prosocial behaviour in the losses context ($\eta_{p}^{2}=0.37$) relative to the gains context ($\eta_{p}^{2}=0.22$), but that the effects of group membership were similar in both contexts ($\eta_{p}^{2}=0.19$ versus $\eta_{p}^{2}=0.12$). There was no significant interaction between group membership and decision setting in either study (study 1, *F*_1,28_=0.40, *p*=0.53; study 2, *F*_1,29_=0.02, *p*=0.90).

**Table 1.** Incidence of prosocial behaviour as a function of condition. Standard deviations indicated in parentheses. Higher numbers indicate greater rates of helping.

	study 1 (gains)			study 2 (losses)		
	ingroup	outgroup	total	ingroup	outgroup	total
public	0.67 (0.26)	0.58 (0.30)	0.62 (0.25)	0.71 (0.30)	0.66 (0.31)	0.69 (0.29)
private	0.57 (0.30)	0.49 (0.26)	0.51 (0.26)	0.61 (0.29)	0.56 (0.29)	0.59 (0.27)
total	0.62 (0.26)	0.52 (0.25)		0.66 (0.28)	0.61 (0.29)

We next ran a GEE analysis that included a regressor indicating the net EV, to test whether the expected value of helping moderated the influence of preferences or reputational concerns on ingroup favouritism. In particular, we were interested in how the expected value of helping was associated with ingroup-favouring social preferences. On a strong version of a social preference-based account, ingroup favouritism is motivated by an analysis that one will receive more pleasure (or utility) from helping the ingroup relative to the outgroup. This suggests that when net EV (operationalized as the expected value of helping, divided by the cost of helping) is large, group membership should not be as important as net EV because the prosocial option is attractive either way. Similarly, when net EV is small, group membership should not be as important because the prosocial choice is not attractive either way. A strong preference-based account might, however, predict a nonlinear interaction between group membership and expected utility such that that ingroup favouritism is most prominent in an intermediate range in which it is attractive to give to an ingroup member, but not to an outgroup member. In contrast, if social preferences more closely approximate social heuristics to cooperate more with ingroup members, then no such interaction should be observed, because participants are likely to apply the social heuristic regardless of the specific costs of helping. In this analysis, our results from the ANOVA were replicated such that in both studies there were significant main effects of group membership (study 1, *χ*^2^=7.66, *p*=0.006; study 2, *χ*^2^=3.92, *p*=0.05) and decision setting (study 1, *χ*^2^=8.39, *p*<0.006; study 2, *χ*^2^=18.01, *p*<0.001). We also looked at the effect of net EV on prosocial decisions and found that (as would be expected on a simple cost/benefit analysis) the more benefit the prosocial action had for the receiver, and the cheaper it was for the participant, the more likely it was that people helped (study 1, *χ*^2^=38.47, *p*<0.001; study 2, *χ*^2^=27.63, *p*<0.001). However, the expected value derived from helping did not moderate the role of social preferences or reputational concerns in ingroup favouritism, as net EV did not interact significantly with group membership or decision setting. Consistent with ingroup-favouring social preferences functioning as a kind of social heuristic, participants were more prosocial to ingroup than outgroup members (and in public rather than private decisions), but there were also lower rates of helping as EV decreased—a pattern that was observed both for gains (figure 3*a*) and losses (figure 3*b*).

Finally, we looked at correlations between rates of helping across the conditions. Rather than looking at ingroup favouritism specifically, we next tested whether people who helped more in public trials were more or less likely to help in private trials. Overall, results revealed that across both studies, participants who helped in one condition were significantly more likely to help in the other conditions too (all *rs*>0.51, all *ps*<0.001). Someone who helped an ingroup member more also helped an outgroup member more, and someone who helped more in public also helped more in private. This coheres with other evidence on prosocial preferences being evident across different situations [51], and suggests that ingroup-favouring prosocial behaviour is not necessarily at odds with prosocial behaviour towards outgroups.

## 7. Discussion

We used a novel experimental paradigm to address two research questions concerning the psychological mechanisms underlying ingroup favouritism. To what extent is ingroup-favouring prosocial behaviour driven by social preferences favouring the outcomes of ingroup members more than outgroup members, relative to strategic concerns concerning reputation management? And does this differ depending on whether the prosocial action is to help another gain something positive, versus to help them avoid something negative?

The first research question we addressed in this paper concerned the extent to which ingroup favouritism is driven by social preferences regarding others’ outcomes versus strategic concerns to preserve a positive reputation. To test this, we looked at the incidence of prosocial behaviour towards ingroup and outgroup members in a minimal group paradigm under conditions of anonymity or not. To the extent that social preferences drive ingroup favouritism, such ingroup-favouring prosocial behaviour should be evident regardless of whether participants’ decisions are seen by others (cf. [14,15]). Here, the critical test is whether ingroup favouritism can be observed even when decisions are private. However, to the extent that reputational concerns are the primary driver of ingroup favouritism, greater prosocial behaviour towards ingroup members should be observed only in public conditions where preservation of a positive reputation is possible (cf. [30]). For this strong version, the critical test is whether ingroup favouritism is observed for anonymous private decisions. Finally, on the weaker reputational concern-based account, ingroup favouritism should be considerably stronger when decisions are public, even if it is still observed in private (cf. [38]).

Our results provide clear evidence against a strong version of the reputation-based explanation of ingroup favouritism favoured by Yamagishi and co-workers [30,31], whereby ingroup favouritism was observed even in minimal groups under conditions of complete anonymity. We found results consistent with social preferences being an important driver of ingroup favouritism: minimal group members helped people from their own group more relative to another, and did so even when decisions were private and so reputation formation was impossible. It is interesting that, in contrast to previous findings [39], these effects of both group and reputation were evident regardless of the economic cost of helping. Such results are consistent with a social preference to help ingroup members serving more as a general social heuristic, rather than a deliberative and controlled weighing up of the utility to be gained from helping an ingroup versus an outgroup member [13,22,23]. While reputational concerns are evidently important for prosocial behaviour generally (as demonstrated by a greater incidence of prosocial behaviour in public conditions), reputational concerns did not appear to drive ingroup favouritism in particular and so ingroup favouritism cannot be solely explained through recourse to reputational concerns.

The second research question we addressed in this paper was a novel investigation of ingroup favouritism in both gains (where one helps another gain something positive) and losses (where one helps another avoid something negative) contexts. While the vast majority of research on prosocial behaviour has used gains contexts, everyday prosocial behaviour often takes the form of helping another avoid losing something and so the extent to which ingroup favouritism would manifest and be driven by the same psychological mechanisms in both gains and losses contexts is an important—and previously unaddressed—topic of study. We found the same pattern of results across both gains and losses contexts: ingroup favouritism was observed, and this was not affected by whether a decision was public or private. We therefore found no evidence of gains or losses contexts having markedly different effects on intergroup prosocial behaviour. We did, however, find that while reputational concerns influenced behaviour in both gains and losses contexts, whether a decision was public or private had a stronger influence—in terms of effect sizes—on prosocial behaviour in a losses context. Therefore, independent of ingroup favouritism, people are more likely to help when decisions are public rather than private, but this is especially so when the prosocial action is to help another avoid losing something.

Our findings have numerous theoretical and practical implications. First, our results highlight that it would be a mistake to consider ingroup-favouring prosocial behaviour as merely a self-interested phenomenon: even in artificial minimal groups under conditions of anonymity, people behave as though they value the outcomes of ingroup members more than outgroup members. Second, that people’s reputational concerns seemed stronger in losses contexts fits with work on the do-no-harm principle [42,43] such that norms of fairness may be more salient in loss contexts and so the costs of publicly violating these norms are stronger. These results constitute the first direct experimental investigation of ingroup favouritism in gains and losses contexts, suggesting interesting new directions of future research in exploring intergroup prosocial behaviour in different contexts. Third, it is of interest that prosocial behaviour towards ingroups was also associated with increased prosocial behaviour towards outgroups, suggesting that general prosocial preferences may not stop at group lines and could be extended towards outgroup members. Future work should explore the possibility that rather than ingroup favouritism being opposed to greater helping overall, social preferences to care more about one’s ingroup could be used to encourage helping of outgroup members. Fourth, and at a practical level, our results suggest that attempting to change people’s preferences, rather than their reputational beliefs, may be a promising method to promote prosocial behaviour in intergroup contexts.

In conclusion, in this paper we conduct a novel investigation of the psychological processes underlying ingroup favouritism and the manifestation of this in both gains and losses contexts. Our results have both theoretical implications regarding the study of both ingroup favouritism in particular and prosocial behaviour more generally, as well as practical implications as to how prosocial behaviour can be encouraged.

## Data accessibility

Data for this paper are deposited online. The influence of social preferences and reputational concerns on intergroup prosocial behaviour in gains and losses contexts: Dryad (http://dx.doi.org/10.5061/dryad.qm83p)
